# Evaluating the Immune Response in Treatment-Naive Hospitalised Patients With Influenza and COVID-19

**DOI:** 10.3389/fimmu.2022.853265

**Published:** 2022-05-19

**Authors:** Jelmer Legebeke, Jenny Lord, Rebekah Penrice-Randal, Andres F. Vallejo, Stephen Poole, Nathan J. Brendish, Xiaofeng Dong, Catherine Hartley, John W. Holloway, Jane S. Lucas, Anthony P. Williams, Gabrielle Wheway, Fabio Strazzeri, Aaron Gardner, James P. R. Schofield, Paul J. Skipp, Julian A. Hiscox, Marta E. Polak, Tristan W. Clark, Diana Baralle

**Affiliations:** ^1^ School of Human Development and Health, Faculty of Medicine, University of Southampton, Southampton, United Kingdom; ^2^ National Institute for Health Research (NIHR) Southampton Biomedical Research Centre, University of Southampton and University Hospital Southampton National Health Service (NHS) Foundation Trust, Southampton, United Kingdom; ^3^ Institute of Infection, Veterinary and Ecological Sciences, University of Liverpool, Liverpool, United Kingdom; ^4^ School of Clinical and Experimental Sciences, Faculty of Medicine, University of Southampton, Southampton, United Kingdom; ^5^ Cancer Sciences Division, Faculty of Medicine, University Hospital Southampton, Southampton, United Kingdom; ^6^ TopMD Precision Medicine Ltd, Southampton, United Kingdom; ^7^ Centre for Proteomic Research, School of Biological Sciences, University of Southampton, Southampton, United Kingdom; ^8^ NIHR Health Protection Research Unit in Emerging and Zoonotic Infections, Liverpool, United Kingdom; ^9^ ASTAR Infectious Diseases Laboratories (ASTAR ID Labs), Agency for Science, Technology and Research (ASTAR) Singapore, Singapore, Singapore; ^10^ Institute for Life Sciences, University of Southampton, Southampton, United Kingdom

**Keywords:** COVID-19, influenza, adaptive, innate, immune response, blood, transcriptome, survival

## Abstract

The worldwide COVID-19 pandemic has claimed millions of lives and has had a profound effect on global life. Understanding the body’s immune response to SARS-CoV-2 infection is crucial in improving patient management and prognosis. In this study we compared influenza and SARS-CoV-2 infected patient cohorts to identify distinct blood transcript abundances and cellular composition to better understand the natural immune response associated with COVID-19, compared to another viral infection being influenza, and identify a prognostic signature of COVID-19 patient outcome. Clinical characteristics and peripheral blood were acquired upon hospital admission from two well characterised cohorts, a cohort of 88 patients infected with influenza and a cohort of 80 patients infected with SARS-CoV-2 during the first wave of the pandemic and prior to availability of COVID-19 treatments and vaccines. Gene transcript abundances, enriched pathways and cellular composition were compared between cohorts using RNA-seq. A genetic signature between COVID-19 survivors and non-survivors was assessed as a prognostic predictor of COVID-19 outcome. Contrasting immune responses were detected with an innate response elevated in influenza and an adaptive response elevated in COVID-19. Additionally ribosomal, mitochondrial oxidative stress and interferon signalling pathways differentiated the cohorts. An adaptive immune response was associated with COVID-19 survival, while an inflammatory response predicted death. A prognostic transcript signature, associated with circulating immunoglobulins, nucleosome assembly, cytokine production and T cell activation, was able to stratify COVID-19 patients likely to survive or die. This study provides a unique insight into the immune responses of treatment naïve patients with influenza or COVID-19. The comparison of immune response between COVID-19 survivors and non-survivors enables prognostication of COVID-19 patients and may suggest potential therapeutic strategies to improve survival.

## Introduction

Previous studies investigating the differences between patients with COVID-19 or influenza on admission to hospital found that both patient groups present with similar systemic inflammation marker levels including C-reactive protein (CRP), white blood cell count, neutrophil count and neutrophil/lymphocyte ratio (1). Once hospitalised, patients with COVID-19 are at a higher risk of developing respiratory distress, pulmonary embolism, septic shock and haemorrhagic strokes, had a longer length of stay in intensive care, and were more likely to require mechanical ventilation compared to patients with influenza (2). The in-hospital mortality was found to be roughly three times higher for COVID-19 compared to influenza (2).

The viral immune response against influenza is well characterised (3), it involves the innate immune system [e.g. macrophages, granulocytes and dendritic cells, which release proinflammatory cytokines and type I interferons (IFN)] to inhibit viral replication, recruit other immune cells to the site of infection, and stimulate the adaptive immune response which consists of a humoral and a cellular mediated immunity, initiated principally by virus-specific antibodies and T cells. Our current understanding indicates that COVID-19 severity and duration are due to a total or early innate immune and IFN response evasion by SARS-CoV-2 (4–7). While patients infected with influenza are able to mount an IFN response (1), which correlates with quicker recovery and decreased disease severity and mortality (8, 9). Similarly, early administration of IFN-beta for COVID-19 patients results in a lowered in-hospital mortality and quicker recovery (10, 11). Pro-inflammatory cytokine expression occurs for a prolonged time in patients with COVID-19 at similar levels with influenza patients (1), with interleukin (IL)-6 and IL-10 (12–14) associated with increased COVID-19 severity, while it has been observed that the presence of antibodies, CD4+ and CD8+ T cells are correlated with a positive patient outcome (15). Therefore, a key question is if an adaptive immune response differs depending on the disease, and whether specific prognostic markers can be identified.

To address this, we first compared a cohort of hospitalised patients infected with influenza virus with an equivalent cohort of SARS-CoV-2 infected patients identified from individuals hospitalised during the first wave of the pandemic and prior to the availability of approved COVID-19 treatments and vaccines. Secondly, we compared individuals who either survived COVID-19 or who succumbed to COVID-19. Both analyses provides us insights to a natural specific antiviral immune response associated with COVID-19, and with COVID-19 survival. Clinical parameters were recorded and peripheral blood, used for RNA sequencing (RNA-seq), were taken at admission to hospital. We aimed to identify distinct patterns of blood transcript abundances and cellular composition to better understand the COVID-19 specific antiviral immune response and to identify a prognostic signature indicative of COVID-19 outcome.

## Materials and Methods

### Recruitment of Patients Positive for SARS-CoV-2 or Influenza Infection

The study was approved by the South Central - Hampshire A Research Ethics Committee (REC): REC reference 20/SC/0138 (March 16^th^, 2020) for the COVID-19 point of care (CoV-19POC) trial; and REC reference 17/SC/0368 (September 7^th^, 2017) for the FluPOC trial. Patients gave written informed consent or consultee assent was obtained where patients were unable to give consent. The studies were prospectively registered with the ISRCTN trial registry: ISRCTN14966673 (COV-19POC) (March 18^th^, 2020), and ISRCTN17197293 (FluPOC) (November 13^th^, 2017).

The COV-19POC study was a non-randomised interventional trial evaluating the clinical impact of molecular point-of-care testing (mPOCT) for SARS-CoV-2 in adult patients. The trial took place during the first wave of the pandemic, from 20th March to 29th April 2020, and prior to the availability of approved COVID-19 treatments. Patients (≥ 18 years old) were recruited from the Acute Medical Unit (AMU), Emergency Department (ED) or other acute areas of Southampton General Hospital when presenting with acute respiratory illness (ARI), or without ARI but suspected SARS-CoV-2 infection, or without ARI and not a suspected COVID-19 case, according to Public Health England guidelines, but where SARS-CoV-2 testing is considered necessary by the clinical team. ARI is defined as an acute upper or lower respiratory illness or an acute exacerbation of a chronic respiratory illness. Patients were excluded who did not meet the inclusion criteria, declined nasal and/or pharyngeal swabbing, consent declined or whom were already recruited to the study in the last 14 days (16). For this comparative study patients were included who were found to be SARS-CoV-2 positive, according to the QIAGEN QIAstat-Dx PCR testing platform with the QIAstat-Dx Respiratory SARS-CoV-2 Panel (17), in the COV-19POC study.

The FluPOC study was a multicentre randomised controlled trial evaluating the clinical impact of mPOCT for influenza in hospitalised adult patients with acute respiratory illness, during influenza season, using the BioFire FilmArray platform with the Respiratory Panel 2.1 (18). The trial took place during influenza seasons over the two winters of 2017/18 and 2018/19. Patients (≥ 18 years old) presenting with ARI, duration less than 10 days prior to admission to hospital, were recruited from the AMU and ED of Southampton General Hospital and Royal Hampshire County Hospital. Patients were excluded when not fulfilling all the inclusion criteria, receiving a purely palliative treatment approach, declining nasal and/or pharyngeal swabbing, consent declined or whom were previously recruited and re-presented after 30 days after hospital discharge (19).

All participants were recruited within the first 24 hours of admission to hospital, and prior to any treatments. Blood samples including whole blood in PAXgene Blood RNA tubes (BRT) (Preanalytix) were collected from 80 SARS-CoV-2 positive patients and 88 influenza positive patients, within 24 hours of enrolment, and stored at -80°C. For both cohorts the demographic and clinical data were collected at enrolment and outcome data from case note and electronic systems. ALEA and BC data management platforms were used for data capture and management.

### Comparison of Baseline Clinical Characteristics

Baseline clinical characteristics of the patient groups were assessed using R (20) (v4.0.2) and RStudio (21) (v1.3.959) for comparisons between COVID-19 versus influenza, and COVID-19 survivors versus non-survivors. Extreme outliers (values < Q1 - 3 interquartile range, or > Q3 + 3 interquartile range) were identified with the R package rstatix (22) (v0.7.0) and removed. Statistical testing was performed including a Shapiro-Wilk test to assess for data normality followed with either an unpaired parametric T-test (Shapiro-Wilk test p-value > 0.05) or an unpaired non-parametric Wilcoxon test (Shapiro-Wilk test p-value < 0.05) for continuous data, or a Chi-square test for categorical data. The R package table 1 (23) (v1.3) was used to plot the baseline clinical characteristics.

### Extraction of RNA From Clinical Samples and Illumina Sequencing

Total RNA was extracted from PAXgene BRT using the PAXgene Blood RNA Kit (PreAnalytix), according to the manufacturer’s protocol at Containment Level 3 in a Tripass Class I hood. Extracted RNA was stored at -80°C until further use. Following the manufacturer’s protocols, total RNA was used as input material into the QIAseq FastSelect–rRNA/Globin Kit (Qiagen) protocol to remove cytoplasmic and mitochondrial rRNA and globin mRNA with a fragmentation time of 7 or 15 minutes. Subsequently the NEBNext^®^ Ultra™ II Directional RNA Library Prep Kit for Illumina^®^ (New England Biolabs) was used to generate the RNA libraries, followed by 11 or 13 cycles of amplification and purification using AMPure XP beads. Each library was quantified using Qubit and the size distribution assessed using the Agilent 2100 Bioanalyser and the final libraries were pooled in equimolar ratios. Libraries were sequenced using 150 bp paired-end reads on an Illumina^®^ NovaSeq 6000 (Illumina^®^, San Diego, USA). Raw fastq files were trimmed to remove Illumina adapter sequences using Cutadapt v1.2.1 (24). The option “−O 3” was set, so that the 3’ end of any reads which matched the adapter sequence with greater than 3 bp was trimmed off. The reads were further trimmed to remove low quality bases, using Sickle v1.200 (25) with a minimum window quality score of 20. After trimming, reads shorter than 10 bp were removed.

### Data QC and Alignment

QC of read data was performed using FastQC (26) (v0.11.9) and compiled and visualised with MultiQC (27) (v1.5). Samples with <20 million total reads were excluded from further analysis. The STAR index was created with STAR’s (28) (v2.7.6a) genome Generate function using GRCh38.primary_assembly. genome.fa and gencode.v34.annotation.gtf (29) (both downloaded from GENCODE), with –sjdbOverhang 149 and all other settings as default. Individual fastq files were aligned using the –twopassMode Basic flag, with the following parameters specified (following ENCODE standard options): –outSAMmapqUnique 60, outFilterType BySJout, –outFilterMultimapNmax 20, –align SJoverhangMin 8, –outFilterMismatchNmax 999, –out FilterMismatchNoverReadLmax 0.04, –alignIntronMin 20, –alignIntronMax 1000000, –alignMatesGapMax 1000000 and all other options as default. For rMATs (30) (v4.1.0) analysis, STAR was run again as before, but with the addition of –alignEndsType EndToEnd. SamTools (31) (v1.8) was used to sort and index the aligned data.

### Systems Immunology-Based Analysis of Blood Transcript Modules

BTM analysis was performed with molecular signatures derived from 5 vaccine trials (32) as a reference dataset, and BTM activity was calculated using the BTM package (32) (v1.015) in Python (33) (v3.7.2) using the normalized counts as input. Module enrichment significance was calculated using CAMERA (34) (v3.46.0). The significance threshold for the linear model was set at FDR 0.05 for the comparison between patients with COVID-19 or influenza.

### Differential Gene Expression Analysis Between Patient Groups

HTSeq (35) (v0.11.2) count was used to assign counts to RNA-seq reads in the SamTools sorted BAM file using GENCODE v34 annotation. Parameters used for HTSeq were –format=bam, –order=pos, –stranded=reverse, –type=exon and the other options were kept at default. EdgeR (36) (v3.30.3) was used for differential gene expression analysis with R (v4.0.2) in RStudio (v1.3.959). Genes with low counts across all libraries were filtered out using the filterByExpr command. Filtered gene counts were normalised using the Trimmed Mean of M -values (TMM) method. A PCA graph was constructed based on all differentially expressed genes to assess sample clustering. Differentially expressed genes were identified, after fitting the negative binomial models and obtaining dispersion estimates, using the exact test and using a threshold criteria of FDR p-value < 0.05 and log2 fold change < -1 and > 1. Genes which were within the threshold criteria were used for ToppGene gene list enrichment analysis, using the default settings, and GO biological process terms.

### Unbiased Gene Co-Expression Analysis

Gene co-expression analysis was performed with BioLayout (37) (v3.4) using a correlation value of 0.95, other settings were kept at default. Clusters were manually assessed to determine gene expression differences depending on for example patient cohort. Gene clusters were subsequently analysed with ToppGene (38) gene list enrichment analysis, using the default settings, and Gene Ontology (GO) (39, 40) biological process terms. The TMM normalised RNA-seq counts were used, together with the clinical phenotype information, for weighted correlation network analysis (WGCNA) with the R package WGCNA (41), using default settings and a power of 3.

### Topological Mapping of Global Gene Patterns

TopMD Pathway Analysis (42) was conducted using the differential transcript abundances identified by differential gene expression analysis, generating a map of the differentially activated pathways between all patients with COVID-19 or influenza. The TopMD pathway algorithm measures the geometrical and topological properties of global differential gene expression embedded on a gene interaction network (43). This enables plotting and measurement of the differentially activated pathways through extrapolation of groups of mechanistically related genes, called TopMD pathways. TopMD pathways possess a natural hierarchical structure and can be analysed for enriched GO terms, by chi-square test.

### *In Silico* Immune Profiling Predicting Immune Cell Levels Between Patient Groups

Relative abundance of 22 immune cell types and their statistical significance was deconvoluted from whole blood using the reference gene signature matrix (LM22) using CIBERSORTx (44). CIBERSORTx analysis was conducted on the CIBERSORTx website (45) using 100 permutations. Immune cell distribution between the groups were compared by Mann–Whitney test.

### Identification of Immune Signatures as a Predictor for COVID-19 Outcome

Transcript to transcript gene co-expression network analysis with BioLayout 3D (v3.4) (Pearson coefficient 0.85, MCL=1.7) assembled 537 genes differentially expressed (EdgeR, FDR < 0.5 and |log2 fold change > 1|) in blood taken on admission between patients with COVID-19 who either survived or died of COVID-19 within 30 days of admission to hospital. Combinations of 100 genes from the top 4 clusters were assessed as predictor variables for outcome using Boosted Logistic Regression, Bayesian Generalised Linear and RandomForest models within SIMON (46) (v0.2.1) installed with Docker (47) (v20.10.2). TMM normalised gene expression data was centred and scaled. Covariant features were removed based on correlation analysis. Samples were randomly split into train:test subsets at the ratio 75%:25%.

## Results

RNA-seq was undertaken for 80 patients with COVID-19 and 88 patients with influenza. Two patients with COVID-19 were identified as outliers and subsequent assessment revealed elevated white blood cell and lymphocyte counts caused by pre-existing chronic lymphocytic leukaemia (**Supplementary Figure 1**). Five patients with influenza failed quality control (QC) (read count < 20M). This left 78 patients with COVID-19, of whom 62 survived and 16 died within 30 days of hospital admission, and 83 patients with influenza.

### Clinical Differences

Baseline clinical characteristics of the patients with COVID-19 or influenza were assessed. No differences in sex or age were detected, however, a higher proportion of patients with influenza were of White British ethnicity (p-value 1.12x10^-05^) and were current smokers (p-value 9.07x10^-05^). Patients with COVID-19 more commonly had hypertension (p-value 1.42x10^-02^), liver disease (p-value 3.63x10^-02^) and diabetes mellitus (p-value 6.44x10^-03^), whilst underlying chronic respiratory disease was more common in patients with influenza (p-value 1.22x10^-03^). Prior to hospital admission patients with COVID-19 had a longer duration of symptoms (p-value 1.17x10^-05^). At hospital admission a higher respiratory rate (p-value 2.79x10^-02^), the administration of supplementary oxygen (p-value 6.81x10^-03^), higher levels of CRP (p-value 1.73x10^-03^) and lymphocytes (p-value 2.76x10^-02^) were all associated with COVID-19 and once admitted a longer length of stay (p-value 5.51x10^-10^) was associated with increased 30 day mortality (p-value 4.42x10^-05^) (**Table 1**).

**Table 1:** Baseline clinical characteristics and outcomes of hospitalised patients with COVID-19 or influenza.

Baseline demographic data			
** **	**COVID-19**	**Influenza**	**P-value**
	**(N = 78)**	**(N = 83)**	
**Sex, n (%)**			
Female	26 (33.3%)	36 (43.4%)	0.252
Male	52 (66.7%)	47 (56.6%)	
**Age (years)**			
Mean (SD)	60.9 (18.0)	57.8 (18.4)	0.367
**Ethnic category (Code 2001), n (%)**			
White - British	47 (60.3%)	79 (95.2%)	1.12×10^-05^
Asian - Indian	3 (3.8%)	0 (0%)	
Black - African	6 (7.7%)	0 (0%)	
Black - Caribbean	2 (2.6%)	0 (0%)	
Other White background	6 (7.7%)	3 (3.6%)	
Other Asian background	13 (16.7%)	0 (0%)	
Mixed	0 (0%)	1 (1.2%)	
Not stated	1 (1.3%)	0 (0%)	
**Current smoking status, n (%)**			
Yes	4 (5.1%)	21 (25.3%)	9.07×10^-05^
No	67 (85.9%)	62 (74.7%)	
Unknown	7 (9.0%)	0 (0%)	
**Symptom duration (days)**			
Median [Min, Max]	7.00 [0, 21.0]	4.00 [1.00, 10.0]	1.17×10^-05^
			
**Comorbidity**			
** **	**COVID-19**	**Influenza**	**P-value**
	**(N = 78)**	**(N = 83)**	
**Hypertension, n (%)**			
Yes	29 (37.2%)	20 (24.1%)	0.0142
Unknown	4 (5.1%)	0 (0%)	
**Cardiovascular disease, n (%)**			
Yes	16 (20.5%)	14 (16.9%)	0.152
Unknown	3 (3.8%)	0 (0%)	
**Renal disease, n (%)**			
Yes	6 (7.7%)	4 (4.8%)	0.141
Unknown	3 (3.8%)	0 (0%)	
**Liver disease, n (%)**			
Yes	3 (3.8%)	0 (0%)	0.0363
Unknown	3 (3.8%)	0 (0%)	
**Diabetes mellitus, n (%)**			
Yes	19 (24.4%)	8 (9.6%)	0.00644
Unknown	3 (3.8%)	0 (0%)	
**Active malignancy, n (%)**			
Yes	6 (7.7%)	6 (7.2%)	0.193
Unknown	3 (3.8%)	0 (0%)	
**Immunosuppressed, n (%)**			
Yes	4 (5.1%)	5 (6.0%)	0.111
Unknown	4 (5.1%)	0 (0%)	
**Other respiratory disease, n (%)**			
Yes	21 (26.9%)	44 (53.0%)	0.00122
Unknown	3 (3.8%)	0 (0%)	
			
Clinical observations			
	COVID-19	Influenza	P-value
	(N = 78)	(N = 83)	
**Heart rate (beats-per-minute)**			
Mean (SD)	97.3 (17.1)	101 (23.0)	0.39
**Systolic blood pressure (mmHg)**			
Mean (SD)	133 (19.9)	132 (23.6)	0.993
**Respiratory rate (breaths-per-minute)**			
Mean (SD)	26.6 (7.73)	23.8 (5.96)	0.0279
**Temperature (Celsius)**			
Mean (SD)	37.4 (1.01)	37.7 (1.13)	0.0822
**Oxygen saturation (%)**			
Mean (SD)	94.3 (3.75)	94.8 (3.41)	0.548
**Supplementary O2, n (%)**			
Yes	37 (47.4%)	21 (25.3%)	0.00681
No	41 (52.6%)	61 (73.5%)	
**National Early Warning Score 2**			
Mean (SD)	5.28 (2.78)	4.79 (2.57)	0.171
			
**Laboratory results**			
** **	**COVID-19**	**Influenza**	**P-value**
** **	**(N = 78)**	**(N = 83)**	** **
White blood cell count (10*9/L)			
Mean (SD)	8.73 (4.29)	8.64 (3.89)	0.913
Neutrophil cell count (10*9/L)			
Mean (SD)	7.06 (4.07)	6.93 (3.67)	0.895
Lymphocyte cell count (10*9/L)			
Mean (SD)	1.01 (0.411)	0.908 (0.541)	0.0276
**C-reactive protein (mg/L)**			
Mean (SD)	131 (110)	80.2 (78.9)	0.00173
			
**Outcomes**			
** **	**COVID-19**	**Influenza**	**P-value**
** **	**(N = 78)**	**(N = 83)**	** **
**Length of stay (days)**			
Mean (SD)	10.5 (9.51)	3.39 (2.92)	5.51×10^-10^
**Died within 30 days after admission, n (%)**			
Yes	16 (20.5%)	0 (0%)	4.42×10^-05^
No	62 (79.5%)	83 (100%)	

Comparisons are given between patients with COVID-19 or influenza for baseline demographic data, patient outcome, clinical observations, laboratory results and known patient comorbidity. Laboratory results were done on peripheral blood taken on admission to hospital. Similarly, clinical observations were recorded on hospital admission. Statistical testing was done with a Shapiro-Wilk test for data normality followed with either an unpaired parametric T-test or an unpaired non-parametric Wilcoxon test for continuous data, or a Chi-square test for categorical data.

An increased 30-day mortality was associated with older patients (p-value 2.58x10^-09^) between COVID-19 survivors and non-survivors. These non-survivors had a shorter duration of symptoms before being admitted to hospital (p-value 5.38x10^-03^) and underlying comorbidities including hypertension (p-value 1.93x10^-03^), cardiovascular disease (p-value 3.97x10^-03^), diabetes mellitus (p-value 2.31x10^-02^) and respiratory disease (p-value 1.06x10^-02^). Laboratory results of blood taken at hospital admission indicated higher levels of white blood cells (p-value 3.83x10^-02^), total protein (p-value 2.5x10^-03^), creatinine (p-value 3.87x10^-02^), alanine aminotransferase (p-value 2.85x10^-02^), troponin (p-value 2.37x10^-04^), tumour necrosis factor α (TNFα) (p-value 1.43x10^-02^), IL-6 (p-value 2.78x10^-03^), IL-8 (p-value 2.24x10^-02^), IL-1β (p-value 3.78x10^-02^) and IL-10 (p-value 7.51x10^-02^) in patients with COVID-19 who died within 30 days after admission to hospital. Higher admission heart rates were seen in survivors compared to non-survivors (p-value 9.27x10^-03^) (**Table 2**).

**Table 2:** Baseline clinical characteristics and outcomes of hospitalised COVID-19 patients: survivors versus non-survivors.

Laboratory results			
** **	**COVID-19 non-survivors**	**COVID-19 survivors**	**P-value**
	**(N = 16)**	**(N = 62)**	** **
**Sex, n (%)**			
Female	7 (43.8%)	19 (30.6%)	0.488
Male	9 (56.2%)	43 (69.4%)	
**Age (years)**			
Mean (SD)	81.6 (10.4)	55.6 (15.6)	2.58×10^-09^
**Ethnic category (Code 2001), n (%)**			
White - British	14 (87.5%)	33 (53.2%)	0.203
Asian - Indian	1 (6.2%)	2 (3.2%)	
Black - African	1 (6.2%)	5 (8.1%)	
Black - Caribbean	0 (0%)	2 (3.2%)	
Other White background	0 (0%)	6 (9.7%)	
Other Asian background	0 (0%)	13 (21.0%)	
Mixed	0 (0%)	0 (0%)	
Not stated	0 (0%)	1 (1.6%)	
**Current smoking status, n (%)**			
Yes	0 (0%)	4 (6.5%)	0.0291
No	12 (75.0%)	55 (88.7%)	
Unknown	4 (25.0%)	3 (4.8%)	
**Symptom duration (days)**			
Median [Min, Max]	2.00 [0, 14.0]	7.00 [0, 21.0]	0.00538
			
**Comorbidity**			
** **	**COVID-19 non-survivors**	**COVID-19 survivors**	**P-value**
	**(N = 16)**	**(N = 62)**	** **
**Hypertension, n (%)**			
Yes	12 (75.0%)	17 (27.4%)	0.00193
Unknown	0 (0%)	4 (6.5%)	
**Cardiovascular disease, n (%)**			
Yes	8 (50.0%)	8 (12.9%)	0.00397
Unknown	0 (0%)	3 (4.8%)	
**Renal disease, n (%)**			
Yes	3 (18.8%)	3 (4.8%)	0.129
Unknown	0 (0%)	3 (4.8%)	
**Liver disease, n (%)**			
Yes	0 (0%)	3 (4.8%)	0.432
Unknown	0 (0%)	3 (4.8%)	
**Diabetes mellitus, n (%)**			
Yes	8 (50.0%)	11 (17.7%)	0.0231
Unknown	0 (0%)	3 (4.8%)	
**Active malignancy, n (%)**			
Yes	3 (18.8%)	3 (4.8%)	0.129
Unknown	0 (0%)	3 (4.8%)	
**Immunosuppressed, n (%)**			
Yes	1 (6.2%)	3 (4.8%)	0.946
Unknown	1 (6.2%)	3 (4.8%)	
**Other respiratory disease, n (%)**			
Yes	9 (56.2%)	12 (19.4%)	0.0106
Unknown	0 (0%)	3 (4.8%)	
			
Clinical observations			
** **	**COVID-19 non-survivors**	**COVID-19 survivors**	**P-value**
	**(N = 16)**	**(N = 62)**	** **
**Heart rate (beats-per-minute)**			
Mean (SD)	87.6 (15.1)	99.9 (16.8)	0.00927
**Systolic blood pressure (mmHg)**			
Mean (SD)	132 (29.8)	133 (16.8)	0.853
**Respiratory rate (breaths-per-minute)**			
Mean (SD)	27.8 (7.57)	26.3 (7.80)	0.337
**Temperature (Celsius)**			
Mean (SD)	37.3 (1.14)	37.4 (0.978)	0.804
**Oxygen saturation (%)**			
Mean (SD)	93.4 (6.12)	94.6 (2.83)	0.643
**Supplementary O2, n (%)**			
Yes	8 (50.0%)	29 (46.8%)	1
No	8 (50.0%)	33 (53.2%)	
**National Early Warning Score 2**			
Mean (SD)	5.40 (2.44)	5.25 (2.88)	0.906
			
Laboratory results			
** **	**COVID-19 non-survivors**	**COVID-19 survivors**	**P-value**
	**(N = 16)**	**(N = 62)**	** **
**Haemoglobin count (g/L)**			
Mean (SD)	128 (21.3)	138 (20.7)	0.144
White blood cell count (10*9/L)			
Mean (SD)	10.4 (4.27)	8.31 (4.23)	0.0383
Platelet count (10*9/L)			
Mean (SD)	231 (83.9)	249 (90.0)	0.38
Neutrophil cell count (10*9/L)			
Mean (SD)	8.73 (4.15)	6.66 (3.98)	0.063
Lymphocyte cell count (10*9/L)			
Mean (SD)	0.900 (0.419)	1.04 (0.409)	0.142
**Sodium level (mmol/L)**			
Mean (SD)	133 (7.01)	136 (3.90)	0.0878
**Potassium level (mmol/L)**			
Mean (SD)	4.15 (0.971)	4.02 (0.473)	0.824
**Urea levels (mmol/L)**			
Mean (SD)	11.6 (5.98)	6.61 (3.32)	0.0025
**Creatinine level (µmol/L)**			
Mean (SD)	128 (66.7)	83.4 (25.2)	0.0387
**Albumin level (g/L)**			
Mean (SD)	33.9 (4.66)	32.8 (4.78)	0.443
**Bilirubin level (µmol/L)**			
Mean (SD)	12.0 (6.06)	11.1 (4.26)	0.965
**Alanine aminotransferase level (units/L)**			
Mean (SD)	37.0 (37.3)	54.1 (43.4)	0.0285
**Alkaline phosphatase level (units/L)**			
Mean (SD)	93.2 (46.0)	95.2 (48.1)	0.922
**Total protein level (g/L)**			
Mean (SD)	72.7 (9.98)	69.9 (6.26)	0.367
**Lactate dehydrogenase level (units/L)**			
Mean (SD)	841 (357)	914 (486)	0.864
**Ferritin level (mmol/L)**			
Mean (SD)	1420 (2020)	974 (794)	0.841
**Troponin level (ng/L)**			
Mean (SD)	164 (194)	9.55 (6.67)	0.000237
**C-reactive protein (mg/L)**			
Mean (SD)	172 (165)	121 (90.7)	0.662
**IL-6 level (pg/ml)**			
Mean (SD)	174 (142)	59.9 (47.8)	0.00278
**TNFa level (pg/ml)**			
Mean (SD)	30.1 (15.6)	19.3 (6.87)	0.0143
**IL-8 level (pg/ml)**			
Mean (SD)	58.6 (29.0)	41.2 (26.5)	0.0224
**IL-1B level (pg/ml)**			
Mean (SD)	0.620 (0.474)	0.378 (0.200)	0.0378
**GM-CSF level (pg/ml)**			
Mean (SD)	2.08 (2.61)	1.48 (0.972)	0.753
**IFNg level (pg/ml)**			
Mean (SD)	35.3 (71.7)	26.6 (55.5)	0.313
**IL-10 level (pg/ml)**			
Mean (SD)	39.5 (36.7)	15.7 (9.35)	0.00181
**IL-33 level (pg/ml)**			
Mean (SD)	0.543 (0.387)	0.340 (0.277)	0.0751
			
**Outcomes**			
** **	**COVID-19 non-survivors**	**COVID-19 survivors**	**P-value**
	**(N = 16)**	**(N = 62)**	** **
**Length of stay (days)**			
Mean (SD)	4.93 (2.34)	11.9 (10.1)	
**Died within 30 days after admission, n (%)**			
Yes	16 (100%)	0 (0%)	<2.00×10^-16^
No	0 (0%)	62 (100%)	

Comparisons are given between COVID-19 survivors and non-survivors for baseline demographic data, patient outcome, clinical observations, laboratory results and known patient comorbidity. Laboratory results were done on peripheral blood taken on admission to hospital. Similarly, clinical observations were recorded on hospital admission. Statistical testing was done with a Shapiro-Wilk test for data normality followed with either an unpaired parametric T-test or an unpaired non-parametric Wilcoxon test for continuous data, or a Chi-square test for categorical data.

### Molecular Differences

The median sequencing depths obtained were: 60.4 million reads for the patients with COVID-19, 58.9 million reads for the patients with influenza (**Supplementary Figure 2A**), 55.7 million reads for the COVID-19 non-survivors and 62.6 million reads for the COVID-19 survivors (**Supplementary Figure 2B**). Clustering of blood transcriptomes revealed homogeneity between patients with COVID-19 or influenza suggesting any variation to be subtle (**Supplementary Figure 3A**), while a partial separation was found between patients who survived or died of COVID-19 indicative of a larger variation (**Supplementary Figure 3B**).

### Contrasting Innate and Adaptive Immune Programmes

**Figure 1:**
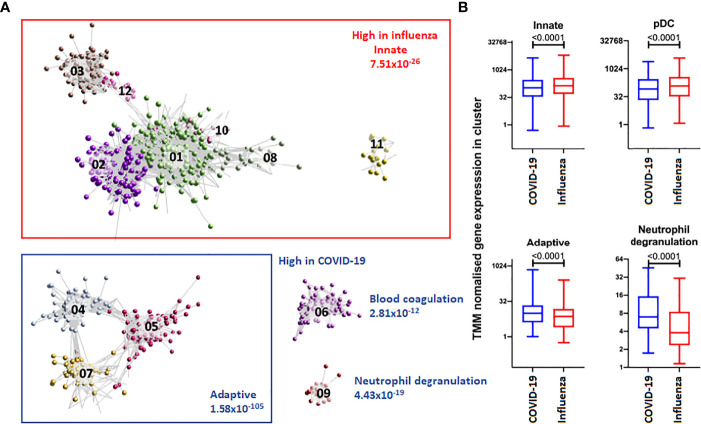
Top 12 clusters identified with BioLayout. **(A)** Enrichment of gene clusters in blood of patients with influenza (annotated in red) and COVID-19 (annotated in blue). Increased abundances of gene transcripts in influenza patients are involved with an innate immune response, while in COVID-19 clusters are involved with an adaptive immune response, blood coagulation and neutrophil degranulation. **(B)** After TMM normalisation a significant difference in gene clusters between patients with influenza or COVID-19 was detected. The abundance of gene transcripts involved with an innate immune response and plasmacytoid dendritic cell were observed to be higher in influenza patients. In contrast, the abundance of gene transcripts involved with an adaptive immune response and neutrophil degranulation was higher in COVID-19 patients.

Analysis of blood transcript modules (BTMs) between patients with COVID-19 or influenza revealed upregulated BTMs in COVID-19 related to the cell cycle and adaptive immune response, primarily CD4+ T cells, B cells, plasma cells and immunoglobulins. In contrast, downregulated BTMs showed signatures associated with monocytes, inflammatory signalling and an innate antiviral and type I IFN response (**Supplementary Figure 4**). Gene co-expression analysis, on a total of 4,093 transcript abundances, between patients with COVID-19 or influenza, identified 50 clusters of four or more genes. These clusters of increased transcript abundances clearly separated patients with COVID-19 from patients with influenza (**Figure 1** and **Table 3**). Gene clusters specific for patients with COVID-19 were involved in adaptive immunity, pointing to activation/priming of T cells and B cells, including induction of proliferation (cluster 4, FDR 3.97x10^-57^), neutrophil degranulation (cluster 9, FDR 4.33x10^-19^) and blood coagulation (cluster 6, FDR 2.84x10^-12^). While gene clusters specific for patients with influenza were involved with innate immunity, including genes expressed in plasmacytoid dendritic cells (cluster 2, FDR 4.17x10^-22^) associated with defence response to virus (cluster 2, FDR 1.34x10^-37^), and genes associated with type 1 helper T cell stimulation (cluster 10, FDR 4.53x10^-03^), dendritic cell morphogenesis (cluster 11, FDR 1.37x10^-02^), and myeloid cell activation (cluster 1, FDR 5.16x10^-13^ and cluster 8, FDR 4.15x10^-04^).

**Table 3:** Summary of the top 12 BioLayout clusters.

Cluster	No. of genes	Cell type	Top biological process	Disease
		(FDR)	(FDR)	
1	362	Myeloid	Cell activation	Influenza
		(1.20x10^-24^)	(5.16x10^-13^)	
2	264	Plasmacytoid dendritic cell	Defence response to virus	Influenza
		(4.17x10^-22^)	(1.34x10^-37^)	
3	166	Erythroblast	Erythrocyte differentiation	Influenza
		(5.31x10^-20^)	(1.70x10^-05^)	
4	140	Progenitor B cell/T cell	Mitotic cell cycle	COVID-19
		(1.28x10^-131^)	(3.97x10^-57^)	
5	100	Progenitor pluripotent cells	Translation	COVID-19
		(1.38x10^-02^)	(8.48x10^-04^)	
6	96	Megakaryocytes/platelets	Blood coagulation	COVID-19
		(3.30x10^-92^)	(2.84x10^-12^)	
7	64	Plasma cells	Response to stress	COVID-19
		(1.27x10^-28^)	(6.41x10^-09^)	
8	29	Myeloid cells	Myeloid leukocyte activation	Influenza
		(2.57x10^-03^)	(4.15x10^-04^)	
9	20	Neutrophils	Neutrophil degranulation	COVID-19
		(1.11x10^-03^)	(4.43x10^-19^)	
10	18	Antigen presenting cells	Th1 stimulation	Influenza
		(2.21x10^-03^)	(4.53x10^-03^)	
11	16	Dendritic cells	Cell morphogenesis	Influenza
		(4.32x10^-04^)	(1.37x10^-02^)	
12	14	Not specified	Histone modification	Influenza
			(3.55x10^-02^)	

Gene clusters were identified with BioLayout (r=0.85, MCL = 1.7). For each cluster the number of genes, predicted cell type and top biological process are given and whether that cluster was enriched in patients with COVID-19 or influenza.

### Topological Mapping of Global Gene Patterns

Topological analysis was used to define a global map of differentially activated pathways between COVID-19 and influenza. The first differentially activated pathway, with peak gene *UBA52*, was associated with cytoplasmic ribosomal proteins (FDR 1.55x10^-146^) and translation factors (FDR 7.90x10^-07^). This pathway was also found to be enriched for genes expressed by transcription factor *Myc* against the ChEA 2016 transcription factor database (FDR 7.07x10^-53^) and of dendritic cells in the ARCHS4 transcription factors’ co-expression database (FDR 1.34x10^-36^). Activated Myc represses *IRF7* and a significantly lower abundance of *IRF7* was found in patients with COVID-19 (**Supplementary Figure 5**). The second differentially activated pathway, with peak gene *NDUFAB1*, was associated with mitochondrial complex I assembly model OXPHOS system (FDR 2.81x10^-66^). The third differentially activated pathway, with peak gene *PSMD14*, was associated with proteasome degradation (FDR 1.46x10^-64^) [**Supplementary Figure 6** with full detail in **Supplementary File 1** and the global map of differentially activated pathways available online (48)].

### Deconvolution of Cell Subsets Supports Innate and Adaptive Immune Response Differences

**Figure 2:**
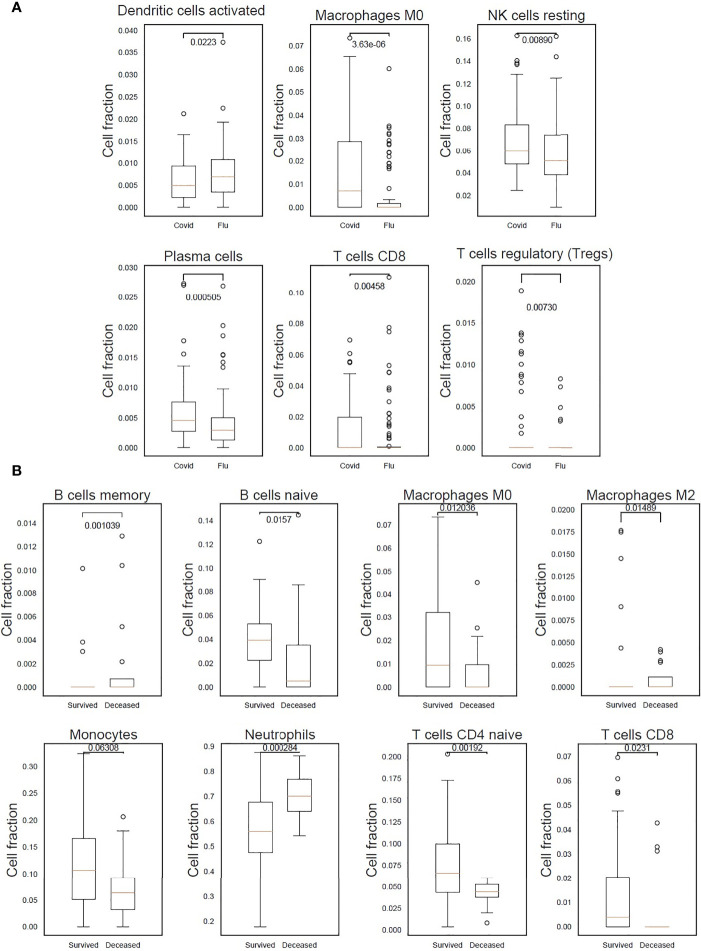
Differences in immune response indicated by predicted cell types in patients with COVID-19, who either survived or died, and patients with influenza. **(A)** M0 macrophages, resting natural killer (NK) cells, plasma cells, cytotoxic CD8+ T cells and regulatory T cells were found to be significantly higher in COVID-19 patients. In influenza patients a significantly higher proportion of activated dendritic cells was detected. **(B)** A statistically significant higher count of neutrophils in COVID-19 patients who died after 30 days indicating the presence of an elevated innate immune response. While an adaptive immune response was detected in COVID-19 survivors as can be seen by the statistically significant higher count of naïve B cells, and CD4+ and CD8+ T cells.

Levels of different predicted immune cell types were assessed between patients with COVID-19 or influenza. Patients with COVID-19 had significantly higher levels of M0 macrophages (p-value 3.63x10^-06^), plasma cells (p-value 5.05x10^-04^), cytotoxic CD8+ T cells (p-value 4.58x10^-03^), regulatory T cells (p-value 7.30x10^-03^) and resting natural killer cell (p-value 8.90x10^-03^). While patients with influenza had significantly higher levels of activated dendritic cells (p-value 2.23x10^-02^) (**Figure 2A** and **Supplementary Figure 7A**). Predicted immune cell type levels between COVID-19 survivors and non-survivors indicated an increase of neutrophils (p-value 2.84x10^-04^) in patients who died of COVID-19 indicative of an elevated innate immune response. In contrast, an increase of naïve CD4+ T cells (p-value 1.92x10^-03^), M0 macrophages (p-value 1.20x10^-02^), M2 macrophages (p-value 1.48x10^-02^), naïve B cells (p-value 1.57x10^-02^) and naïve cytotoxic CD8+ T cells (p-value 2.31x10^-02^), were identified in patients who went on to survive COVID-19 indicative of an adaptive immune response (**Figure 2B** and **Supplementary Figure 7B**).

### Adaptive Immune Response Associates With Patient Survival in COVID-19

**Figure 3:**
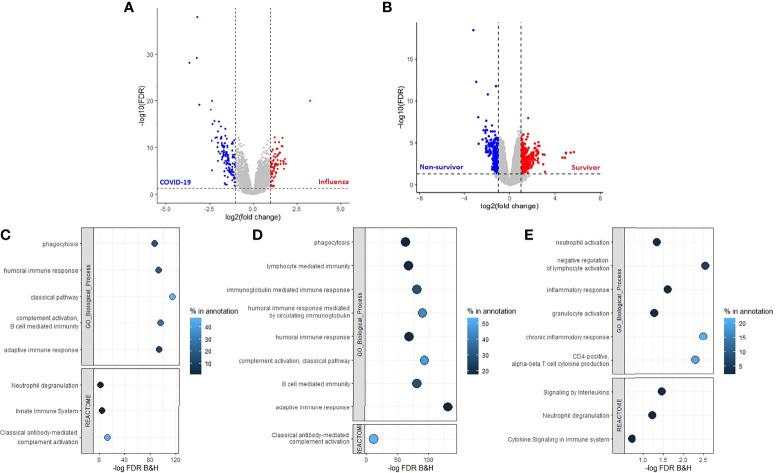
Adaptive immune response associated with COVID-19 and a positive patient outcome. Volcano plots **(A)** between patients with COVID-19 or influenza and **(B)** between COVID-19 survivors and non-survivors, threshold criteria used FDR < 0.05 and log2 fold change < -1 or >1, transcript which met criteria were used for enrichment analysis with ToppGene. **(C)** Enrichment analysis of the transcripts with an increased abundance in patients with COVID-19 identified an increased adaptive immune response which was also detected in **(D)** patients with COVID-19 who were still alive 30 days after hospital admission. **(E)** Increased innate immune response in patients who died of COVID-19 after 30 days of hospital admission. Percentage in annotation is the ratio of the input query genes overlapping with the genes in the pathway annotation.

After filtering out transcripts with low counts a total of 20,542 gene transcript abundance measures were obtained between patients with COVID-19 or influenza, and 23,850 gene transcript abundance measures between COVID-19 survivors and non-survivors. After further filtering (FDR < 0.05, log2 fold change < -1 or > 1) the following number of transcripts were found at a higher abundance: 71 transcripts in patients with influenza, 126 transcripts in patients with COVID-19 (**Figure 3A** and **Supplementary File 2**), 265 transcripts in COVID-19 survivors and 272 transcripts in COVID-19 non-survivors (**Figure 3B** and **Supplementary File 3**). The transcripts with increased abundance in patients with COVID-19 were associated with humoral immune response, complement activation and B cell mediated immunity (**Figure 3C**), and the majority of these COVID-19 specific transcripts (83/126) were immunoglobulin genes, associated with an adaptive immune response, and were present at a higher abundance in primarily patients with COVID-19 (**Supplementary Figure 8**). This adaptive immune response, including complement activation, B cell mediated immunity and a humoral immune response mediated by circulating immunoglobulins, was associated specifically with COVID-19 survivors (**Figure 3D**). While the transcripts specific for COVID-19 non-survivors were associated with an inflammatory response including interleukin signalling, neutrophil activation and neutrophil degranulation (**Figure 3E**).

### Clinical Covariates and Their Correlation With the Abundance of Different Gene Transcript Clusters

Weighted gene co-expression network analysis (WGCNA) identified 23 modules of co-expressed gene transcripts, and these were assessed with GO analysis to identify the associated biological processes terms. Furthermore, the correlation between these gene transcripts modules and the known clinical covariates was determined to investigate the potential drivers of the differences in gene transcript abundances (**Supplementary Figure 9**). The gene module which had the highest positive correlation (0.51, p-value 3x10^-12^) with the type of viral infection, was found to be involved with complement activation *via* the classical pathway. This gene module was characterised by a weaker positive correlation (0.34, p-value 1x10^-05^) with the duration of symptoms before hospital admission, lymphocyte count (0.33, p-value 2x10^-05^), and a negative correlation with the presence of other underlying chronic respiratory disease (-0.37, p-value 1x10^-06^). Additionally, B cell activation was negatively correlated with e.g. age (-0.32, p-value 4x10^-05^) and death within 30 days of hospital admission (-0.25, p-value 1x10^-03^). While neutrophil degranulation and myeloid leukocyte activation were positively correlated among others with oxygen supplementation (r=0.26, p-value 1x10^-03^), and death within 30 days of admission (r=0.25, p-value 1x10^-03^) respectively. The type of viral infection was furthermore the biggest driver for differences in blood coagulation (r=0.39, p-value 2x10^-07^), cellular response to interleukin-13 (r=0.38, p-value 5x10^-07^). In contrast, positive regulation of chemokine production was negatively correlated with the type of viral infection (r=-0.28, p-value 3x10^-04^) (**Table 4**).

**Table 4:** Clinical covariates and their correlation with different gene transcript clusters.

GO biological process (FDR)	No. of genes	Negative correlation	Positive correlation
		(R value < -0.20, p-value)	(R value > 0.20, p-value)
Complement activation (classical pathway) (1.48x10^-65^)	63	Other underlying chronic respiratory disease (-0.37, 1x10^-06^)	Viral infection (0.51, 3x10^-12^)
			Symptom duration (days) (0.34, 2x10^-05^)
			Lymphocyte count (0.33, 2x10^-05^)
B cell activation (2.40x10^-09^)	13	Other underlying chronic respiratory disease (-0.34, 1x10^-05^)	Lymphocyte count (0.36, 2x10^-06^)
		Age (-0.32, 4x10^-05^)	
		Neutrophil count (-0.28, 3x10^-04^)	
		Died within 30 days of admission (-0.25, 1x10^-03^)	
		White blood cell count (-0.23, 4x10^-03^)	
Neutrophil degranulation (1.27x10^-18^)	53		C-reactive protein level (0.54, 1x10^-13^)
			Neutrophil count (0.47, 4x10^-10^)
			White blood cell count (0.45, 3x10^-09^)
			O2 supplementation (0.26, 1x10^-03^)
Myeloid leukocyte activation (3.66x10^-21^)	54	Lymphocyte count (-0.25, 1x10^-03^)	Neutrophil count (0.47, 2x10^-10^)
			White blood cell count (0.41, 6x10^-08^)
			C-reactive protein level (0.34, 1x10^-05^)
			Died within 30 days of admission
			(0.25, 1x10^-03^)
Positive regulation of chemokine production (6.85x10^-04^)	6	Type of viral infection (-0.28, 3x10^-04^)	Other underlying respiratory disease (0.46, 9x10^-10^)
Blood coagulation (1.78x10^-22^)	55		Type of viral infection (0.39, 2x10^-07^)
			Symptom duration (days) (0.27, 6x10^-04^)
Cellular response to interleukin-13 (1.88x10^-02^)	2	Other underlying respiratory disease(-0.32, 5x10^-06^)	Type of viral infection (0.38, 5x10^-07^)
		White blood cell count (-0.35, 5x10^-06^)	Symptom duration (days) (0.25, 1x10^-03^)
		Neutrophil count (-0.38, 6x10^-07^)	

Weighted correlation network analysis was performed to assess the correlation between different clinical covariates, given are the correlation values and the p-values, and the expression of specific gene transcript clusters. These gene transcript clusters underwent GO analysis which revealed the associated biological process which is given together with the FDR p-value, and the number of genes from the input.

### Immune Signatures as Predictors of COVID-19 Outcome

**Figure 4:**
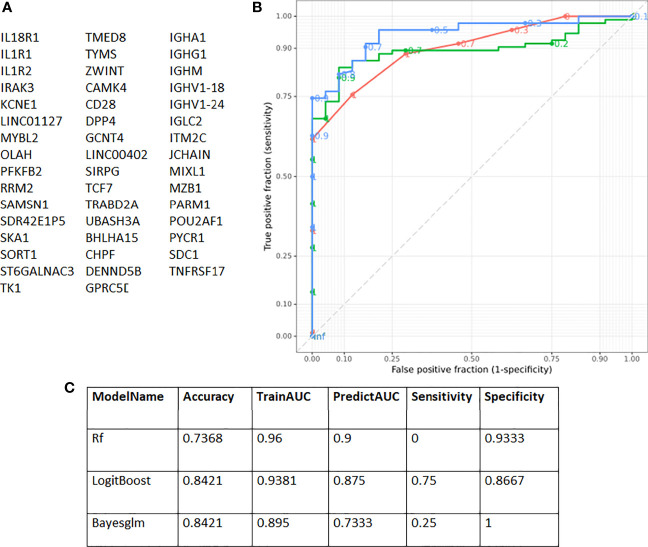
Receiver Operating Characteristic (ROC) curves showing prediction accuracy COVID-19 survivors and non-survivors. **(A)** Genes identified with EdgeR and gene co-expression analysis and used for subsequent modelling. **(B)** ROC curves according to the three models used [Boosted Logistic Regression (LogitBoost), Bayesian Generalised Linear (Bayesglm) and RandomForest (rf)]. **(C)** In total three different models were used [RandomForest (rf), Boosted Logistic Regression (LogitBoost) and Bayesian Generalised Linear (Bayesglm)]. The 47 genes identified with gene co-expression and differential gene expression analysis were used as input. The highest sensitivity obtained was 75% and for specificity 93%.

A distinct immune signature was selected and assessed for prediction accuracy in stratifying patients with COVID-19 for disease outcome. This signature consists of 47 genes (**Figure 4A**), representative of the four biggest gene clusters associated with COVID-19 survival or fatality. These gene clusters are associated with humoral immune response mediated by circulating immunoglobulin (FDR p-value 2.23x10^-46^), nucleosome assembly (FDR p-value 5.46x10^-19^), regulation of T-helper 1 cell cytokine production (FDR p-value 4.24x10^-03^) and regulation of T cell activation (FDR p-value 4.51x10^-04^) (**Supplementary Figure 10**). This gene signature was highly predictive for outcome, with a maximum specificity of 75% and sensitivity of 93% (**Figures 4B, C**).

## Discussion

This study demonstrated important immune differences between hospitalised adults with COVID-19 and influenza and between COVID-19 survivors and non-survivors, using samples taken from COVID-19 patients obtained in the first SARS-CoV-2 wave, and prior to the use of treatments and vaccines.

Known COVID-19 prognostic mortality and severity variables (49) were compared between patients with COVID-19 or influenza. We found more active smokers and underlying respiratory disease among influenza patients. Among patients with COVID-19 a higher CRP [which has previously been reported to be similar upon admission to hospital between patients with COVID-19 or influenza (1)], and a higher proportion of patients with hypertension, liver disease [which has been classified as a low or very low certainty predictor (49)], and diabetes was found compared to those with influenza. Similar to what has been previously reported (1) upon admission to hospital both patients with COVID-19 or influenza presented with similar white blood cell and neutrophil counts, and although we detected a difference in lymphocytes between patients with COVID-19 or influenza, there was no difference in the neutrophil/lymphocyte ratio. Similar to Piroth et al. (2), we found that the average length of stay was higher for patients with COVID-19 and more patients with COVID-19 needed supplementary oxygen compared to influenza. Piroth et al. (2) previously reported a roughly three times higher relative risk of death for COVID-19 however in our cohort no influenza patients died whilst admitted to hospital and so this could not be assessed. As reported, we found that high certainty prognostic variables for mortality and/or severity of increased age, hypertension, cardiovascular disease, diabetes, underlying respiratory disease and high white blood cell levels (49) in COVID-19 non-survivors. Here we also report the findings of an increased heart rate in COVID-19 survivors, but further research is needed to confirm that this is independently associated with survival. While it has previously been reported that CRP and neutrophil/lymphocyte ratio were elevated in critically ill patients with COVID-19 (1), we detected no difference in CRP, neutrophil count and lymphocyte count between COVID-19 survivors and non-survivors.

Several differentially activated gene pathways were detected between COVID-19 and influenza. One differentially activated pathway was enriched for genes related to ribosomal pathways indicating the possible impact on translational machinery. Furthermore, the pathway was enriched for genes transcribed by Myc. Activated Myc represses *IRF7* which regulates type I IFN production (50), and correspondingly a significant lower *IRF7* expression and a lower IFN response was detected in patients with COVID-19. This impaired IFN response in COVID-19 may be due to the virus avoiding or delaying an intracellular innate immune response to type I and type III IFNs (4–7). A pathway involved with the mitochondrial complex I assembly model OXPHOS system was differentially activated supporting reported increased COVID-19 severity due to SARS-CoV-2 being able to highjack and disrupt mitochondrial dynamics of immune cells (51). Cellular ubiquitin-proteasome pathways which are known to play important roles in coronavirus infection cycles were found to be differentially activated (52), these pathways might reflect increased viral replication and suppression of host IFN signalling pathways, including increased degradation of IκBα which suppresses the IFN-induced NF-κB activation pathway. However, *PSMD14* the peak marker of this pathway prevents *IRF3* autophagic degradation and therefore permits IRF3-mediated type I IFN activation (53).

An impaired immune response to viruses and IFN signalling in patients with COVID-19 was detected, as previously reported (4–7), compared to patients with influenza, which are known to produce strong IFN responses (1). Furthermore, in accordance with evidence of aberrant blood clotting in COVID-19 (54, 55), transcripts expressed by megakaryocytes and platelets associated with blood coagulation were at a higher abundance in COVID-19 patients. Innate immune response related gene pathways were found to be associated with influenza, and an adaptive immune response and an increase of a wide range of immunoglobulin transcripts for patients with COVID-19, which is consistent with previous findings (56). This adaptive immune response was found to have a stronger positive correlation with the type of viral infection as opposed to the difference in duration of symptoms between the patients before admission to hospital. An increase in gene pathways involved with an adaptive immune response and increase in predicted CD4+ and CD8+ T cells and naïve B cells was detected and associated with young COVID-19 survivors, highlighting the importance of an efficient adaptive immune response as previously reported (15). Predicted naïve CD4+ T cells were higher compared to predicted CD8+ T cells indicating an increased CD4+ T cell response to SARS-CoV-2, supporting previous observations (15, 57), which has been found to control primary SARS-CoV-2 infection (58). Predicted CD8+ T cells were mostly seen in COVID-19 survivors which has been associated with a positive COVID-19 outcome (58, 59).

An enrichment of pathways involved with the negative regulation of lymphocyte activation and increased neutrophil activation and degranulation, a significant decrease in predicted naïve B cells and naïve CD4+ and CD8+ T cells, and an increase of the neutrophil cells was detected in COVID-19 non-survivors. Similar to previous studies reporting elevated neutrophil levels in blood (60) and lungs (61–64) in severe COVID-19. The activation and degranulation of neutrophils were positively correlated with patients receiving oxygen supplementation and who eventually died within 30 days of hospital admission. Additionally, gene pathways associated with inflammatory response and cytokine signalling, a higher transcript abundance of several IL genes (*IL1-RAP*, *IL-10*, *IL1-R1*, *IL1-R2*, *IL18-R1* and *IL18-RAP*) and increased levels of TNFα, IL-1β, IL-8, IL-33, IL-6 and IL-10 in blood were detected in COVID-19 non-survivors. This is similar to findings of positive regulation of genes encoding the activation of innate immune system, viral and IFN response (1), increase of proinflammatory macrophages (65) and elevated IL-6 and IL-10 in severe COVID-19 cases (12–14).

It appears that, and as Sette and Crotty (66) summarised, that COVID-19 severity is largely due to an early virus-driven evasion of innate immune recognition leading to a delayed adaptive immune response with a fatal COVID-19 outcome, as shown by Lucas et al. (67), where the innate immune response is ever-expanding due to an absence of a rapid T cell response. In accordance with a delayed T cell response, we noticed a decrease of dendritic cells in patients with COVID-19 potentially leading to impaired T cell priming. A delayed adaptive immune response can occur in the elderly due to a scarcity of naïve T cells caused by aging (68–70) placing them at an increased risk of death (58). The association of age and COVID-19 severity is already known, for example, as of April 15^th^ 2021 in the United States 95.4% of COVID-19 deaths occurred in 50-year-olds and older, and 59.3% in 75-year-olds and older (71). In our cohort, patients who survived COVID-19 were younger, had a longer duration of symptoms before admission to hospital and higher levels of predicted naïve CD4+ T cells and naïve B cells.

Taken together, in this comparative study we implemented a variety of different bioinformatic analyses on whole blood RNA-seq between a cohort of patients infected with SARS-CoV-2, during the first wave of the COVID-19 pandemic, and patients infected with influenza, with samples taken before treatments for both groups. An increased innate immune response was found to be associated with patients infected with influenza, while an increased adaptive immune response was associated with patients infected with SARS-CoV-2. This early increased adaptive immune response was indicative of patient survival, thus illustrating the importance of an adequate adaptive immune response in successfully countering SARS-CoV-2 infection, while an increased proinflammatory response was seen in COVID-19 non-survivors. Distinct prognostic immune signature genes were identified in whole blood from untreated patients infected with SARS-CoV-2 which can used upon patient admission to hospital to differentiate between COVID-19 patients likely to survive or not.

## Limitations

The authors acknowledge that the inherent characteristics of the dataset being a moderate sample size, sampling time differences between symptom onset and admission to hospital, underlying comorbidities, and the retrospective design could have a direct impact upon the range of immune signature differences observed. However, the gene clusters identified with an adaptive immune response was primarily positively correlated with the type of viral infection, and was weaker correlated to the duration of symptoms before admission to hospital. The comparison between patients with influenza versus patients whom survived COVID-19 was outside the current study’s analytical framework and future work could be directed in this direction.

## Data Availability Statement

The datasets presented in this study can be found in online repositories. The names of the repository/repositories and accession number(s) can be found below: European Genome-Phenome Archive, EGAS00001005971.

## Ethics Statement

The studies involving human participants were reviewed and approved by South Central Hampshire A Research Ethics Committee. The patients/participants provided their written informed consent to participate in this study.

## Author Contributions

TC and DB conceptualized the study. SP and NB screened and recruited the patients and collected the data in the FluPOC and CoV-19POC trials. RP-R and CH sample processing and experiments. JLe, JLo, RP-R, AV, XD, FS, AG, JS, JHi and MP performed data analysis. JLe, JLo, RP-R, AV, FS, AG, JS, JHi and MP drafted the article, and editing by JLe, JLo, RP-R, SP, NB, GW, JS, PS, JHi, MP, TC and DB. Project advice was given by JHo, JSL, JHi, MP and TC. All authors read and approved the final manuscript.

## Funding

The author(s) disclosed receipt of the following financial support for the research, authorship, and/or publication of this article: the CoV-19POC trial was funded by University Hospital Southampton Foundation Trust (UHSFT) and the FluPOC trial by the National Institute for Health Research (NIHR) Post-Doctoral Fellowship Programme. In addition, the CoV-19POC and FluPOC trials were supported by the NIHR Southampton Clinical Research Facility and NIHR Southampton Biomedical Research Centre (BRC). JLe was supported by a PhD studentship from the NIHR Southampton BRC (no. NIHR-INF-0932). RP-R was supported by a PhD studentship from the Medical Research Council Discovery Medicine North Doctoral Training Partnership (no. MR/N013840/1). NB was supported by the NIHR Clinical Lecturer scheme. JHi, CH and XD were supported by the US Food and Drug Administration (no. 75F40120C00085), and this work was partly supported by U.S. Food and Drug Administration Medical Countermeasures Initiative (no 75F40120C00085) awarded to JHi. MP was supported by a Sir Henry Dale Fellowship from Welcome Trust and The Royal Society (no. 109377/Z/15/Z). TC was supported by a NIHR Post-Doctoral Fellowship (no. 2016-09-061). DB and her laboratory are supported by a NIHR Research Professorship (no. RP-2016-07-011). The funders had no role in study design, data collection and analysis, decision to publish, or preparation of the manuscript.

## Conflict of Interest

TC has received speaker fees, honoraria, travel reimbursement, and equipment and consumables free of charge for the purposes of research from BioFire diagnostics LLC and BioMerieux. TC has received discounted equipment and consumables for the purposes of research from QIAGEN. TC has received consultancy fees from Biofire diagnostics LLC, BioMerieux, Synairgen research Ltd, Randox laboratories Ltd and Cidara therapeutics. TC has been a member of advisory boards for Roche and Janssen and has received reimbursement for these. TC is member of two independent data monitoring committees for trials sponsored by Roche. TC has previously acted as the UK chief investigator for trials sponsored by Janssen. TC is currently a member of the NHSE COVID-19 Testing Technologies Oversight Group and the NHSE COVID-19 Technologies Validation Group. JS is a founding director, CEO, employee and shareholder in TopMD Precision Medicine Ltd. FS is a founding director, CTO, employee and shareholder in TopMD Precision Medicine Ltd. PS is a founding director, employee and shareholder in TopMD Precision Medicine Ltd. AG is an employee and shareholder in TopMD Precision Medicine Ltd.

The remaining authors declare that the research was conducted in the absence of any commercial or financial relationships that could be construed as a potential conflict of interest.

## Publisher’s Note

All claims expressed in this article are solely those of the authors and do not necessarily represent those of their affiliated organizations, or those of the publisher, the editors and the reviewers. Any product that may be evaluated in this article, or claim that may be made by its manufacturer, is not guaranteed or endorsed by the publisher.
