# Supplementary material for: Evaluating the Immune Response in Treatment-Naive Hospitalised Patients With Influenza and COVID-19
Source: Front Immunol. 2022 May 19;13:853265. doi: 10.3389/fimmu.2022.853265 (PMC9160963; doi:10.3389/fimmu.2022.853265)
Supplement: Supplementary file 1 [file DataSheet_1.docx]

# **Supplementary Figures**


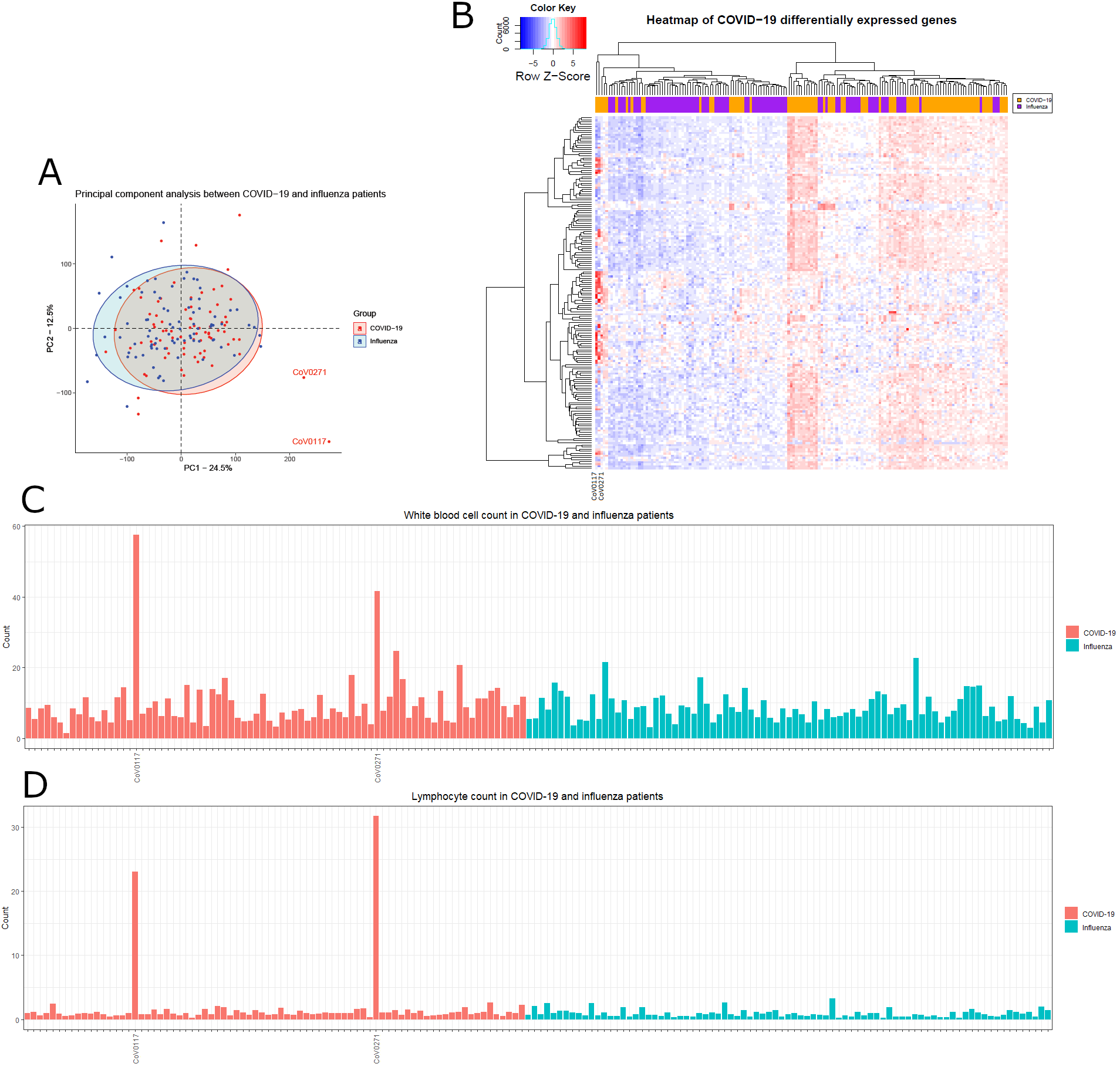


**Supplementary figure 1: Initial sample comparison revealed two patients with COVID-19 which were subsequently excluded in further analyses.** A) Principal Component Analysis (PCA) graph comparing the COVID-19 patients with the influenza patients revealed two COVID-19 outliers (CoV0117 and CoV0271). B) Heatmap comparing the differentially expressed genes found specifically in COVID-19 patients indicates a different expression profile for CoV0117 and CoV0271 compared to the other samples. Assessment of the blood results indicates a high white blood cell count (C) and high lymphocyte count (D) in CoV0117 and CoV0271. Subsequent, analysis of the patient metadata revealed that these samples are from two patients with known chronic lymphocytic leukaemia.


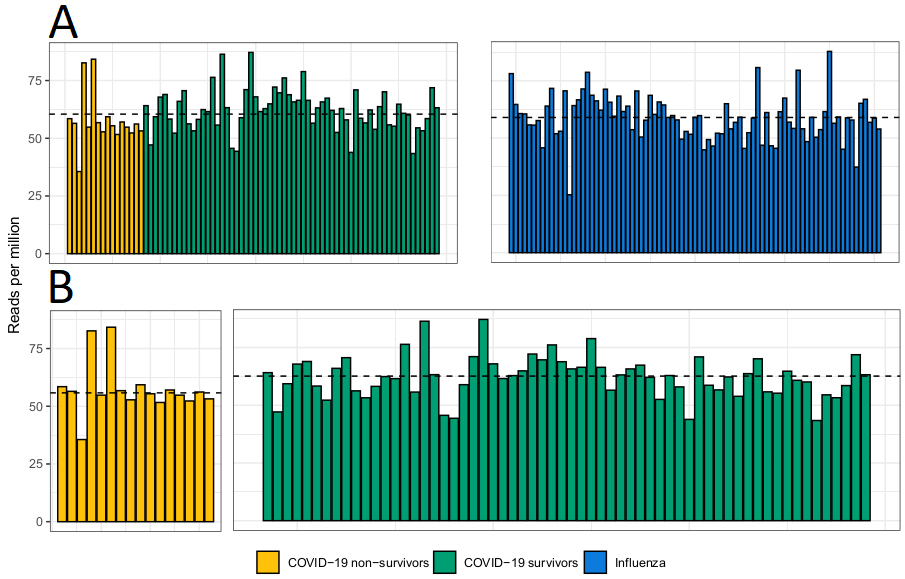


**Supplementary figure 2: Sequence read depths obtained in the patients with COVID-19 or influenza in reads per million.** A) Comparison of the sequence read depths obtained in the 78 patients with COVID-19 (median sequence depth of 60.4 million reads) and 83 patients with influenza (median sequence depth of 58.9 million reads). B) Sequence read depths in the patients with COVID-19 used for the comparison between survivors (median sequence depth of 62.6 million reads) and non-survivors (median sequence depth of 55.7 million reads). Median sequence depths are illustrated by the black dashed line.


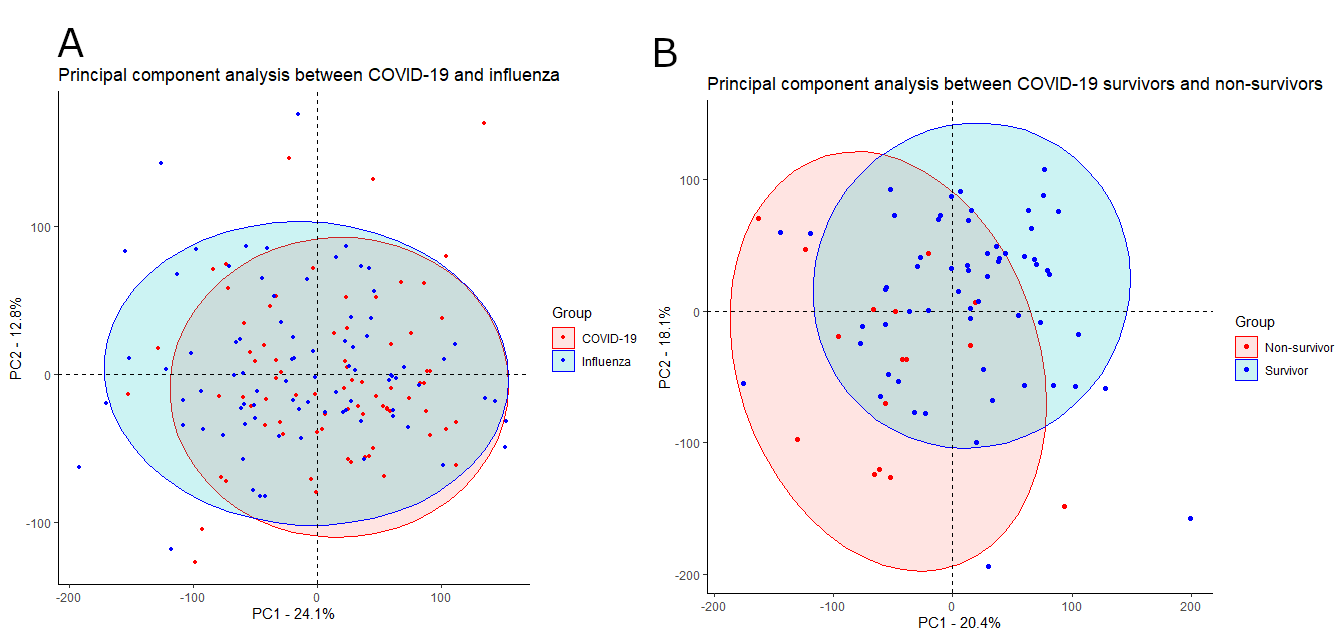


**Supplementary figure 3: Principal component analysis (PCA) between patients with COVID-19 or influenza, and who survived or died of COVID-19.** A) PCA analysis between patients with COVID-19 or influenza showing a substantial overlap between the two different group blood transcriptomes. B) A partial overlap of blood transcriptomes between patients who survived or who died of COVID-19.


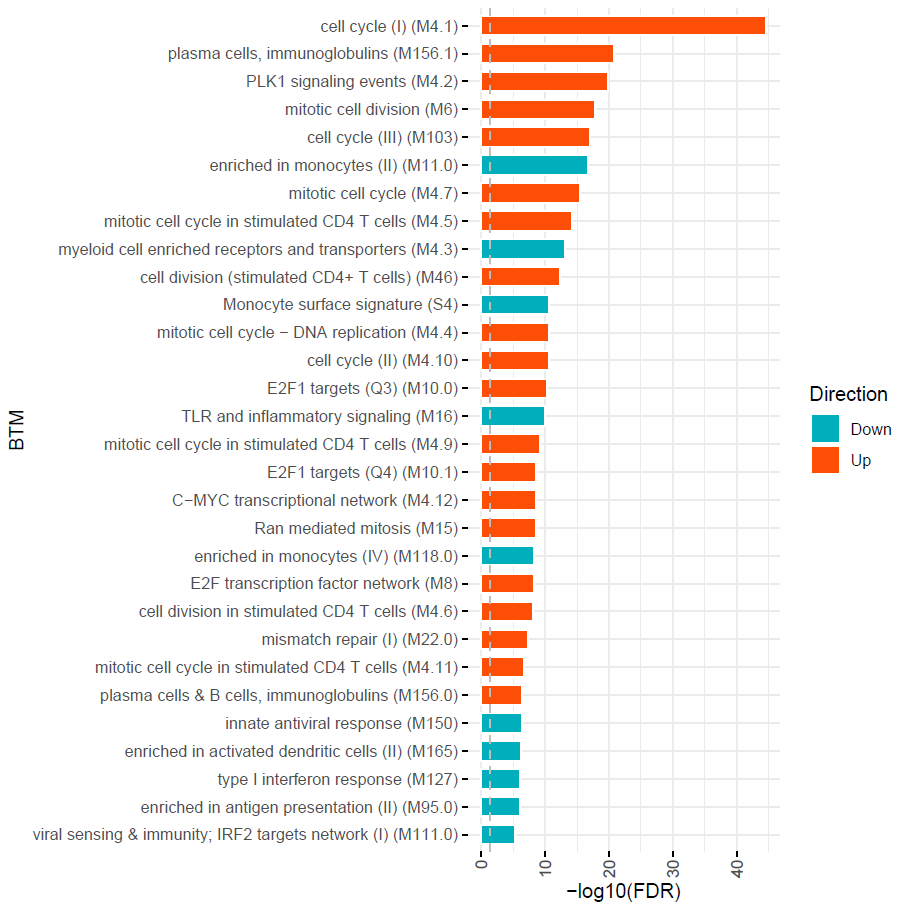


**Supplementary figure 4: Blood transcript module analysis between patients with COVID-19 or influenza.** Upregulated signatures in COVID-19 patients are associated with cell cycle and an adaptive immune response, primarily CD4+ T cells, B cells, plasma cells and immunoglobulins. While the downregulated signatures, associated with influenza patients, are involved with monocytes, inflammatory signalling and an innate antiviral and type I interferon response.


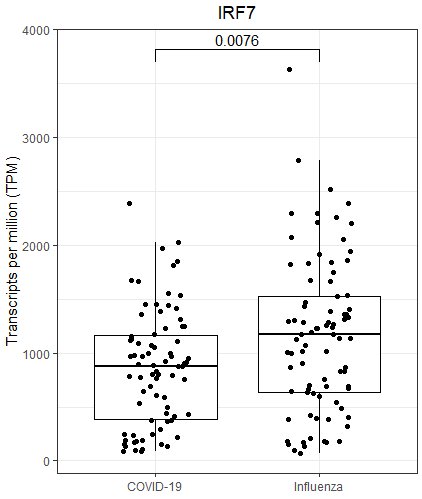


**Supplementary figure 5: Significant difference in IRF7 abundancy between patients with COVID-19 or influenza.** The mean abundancy in patients with influenza is 1162 transcripts per million (TPM). This is significantly lower in patients with COVID-19 at 861 TPM (p-value 7.60·10^-03^). Statistical testing done with Wilcoxon test.


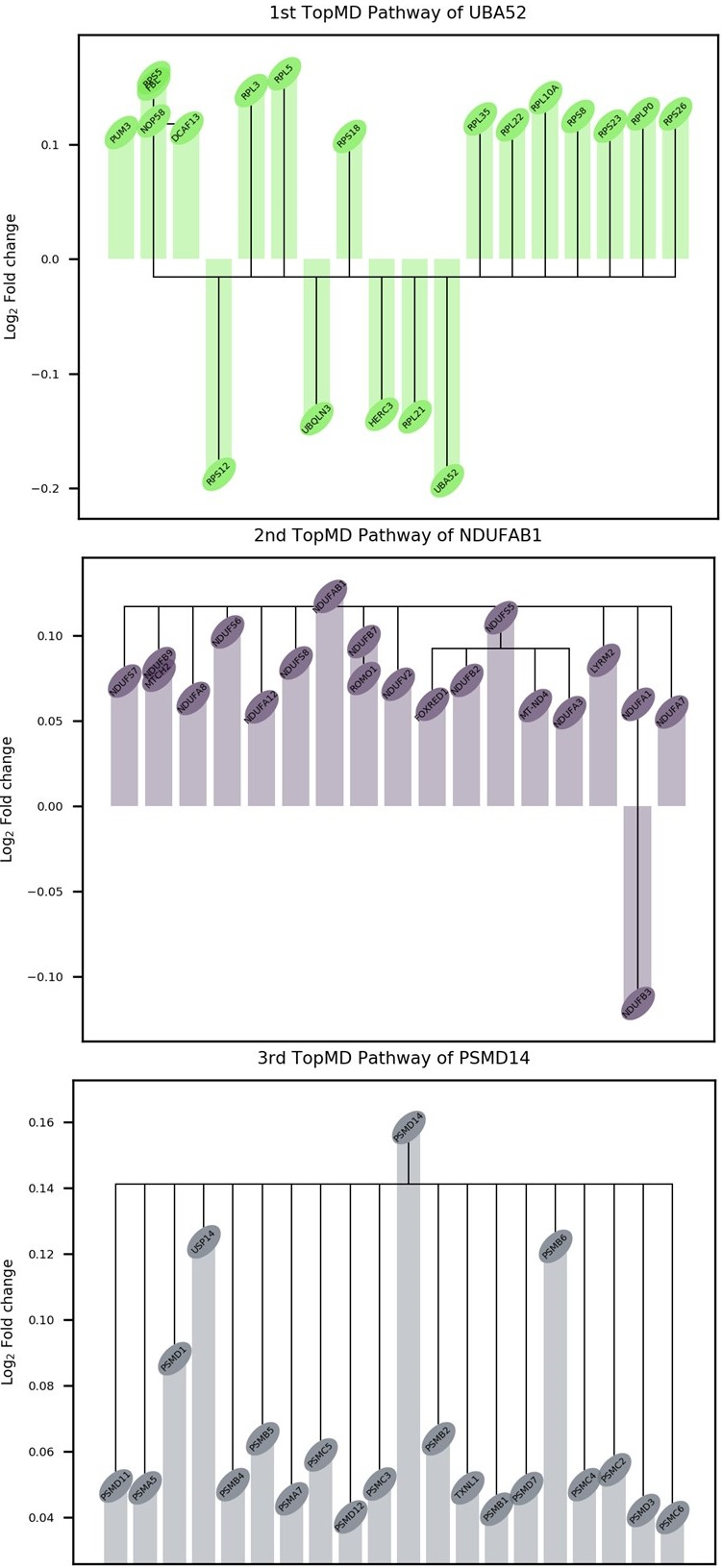


**Supplementary figure 6: Differentially activated pathways between hospitalised patients with COVID-19 or influenza identified with topological analysis.** The difference (Log2 fold change) in patients with COVID-19 compared to patients with influenza is plotted for the top 20 genes of the 1^st^, 2^nd^ and 3^rd^ TopMD pathways.


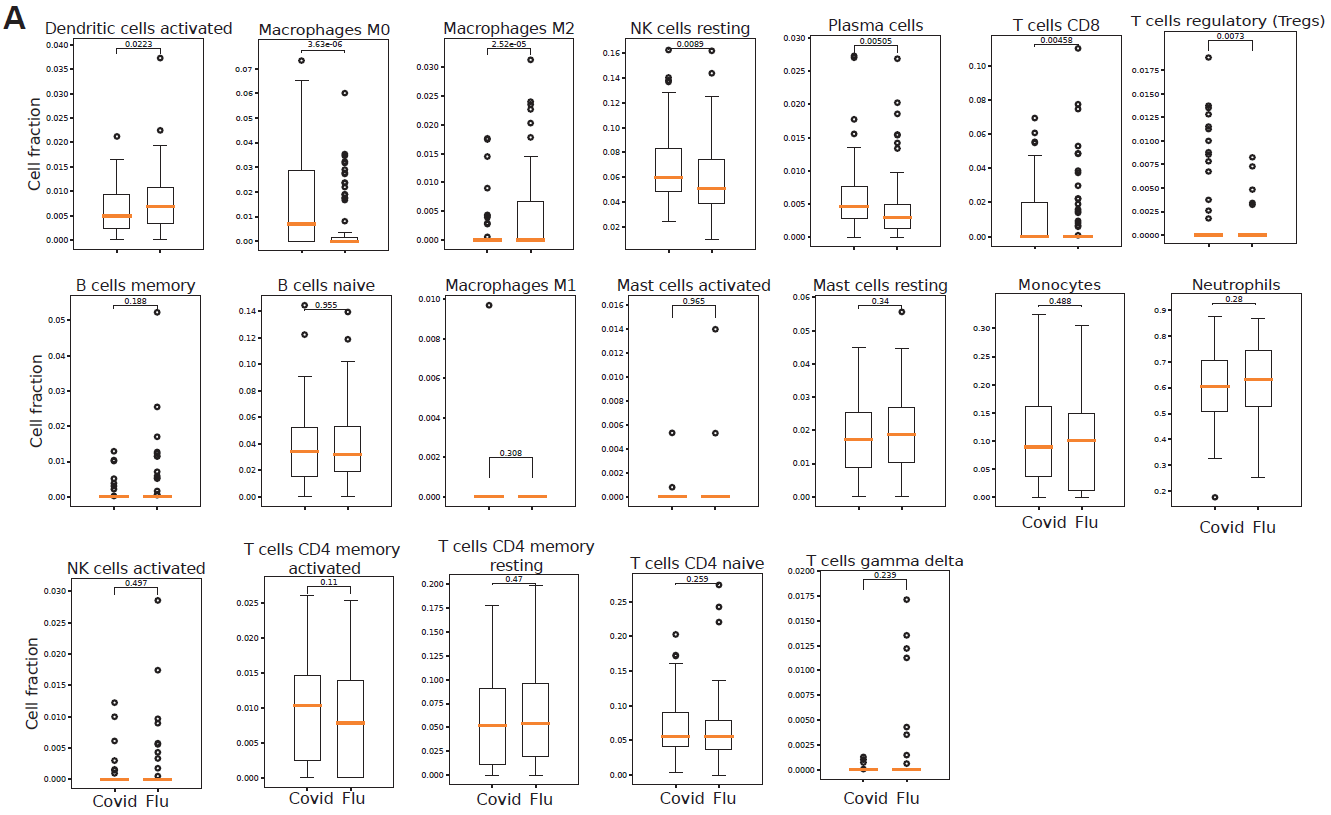


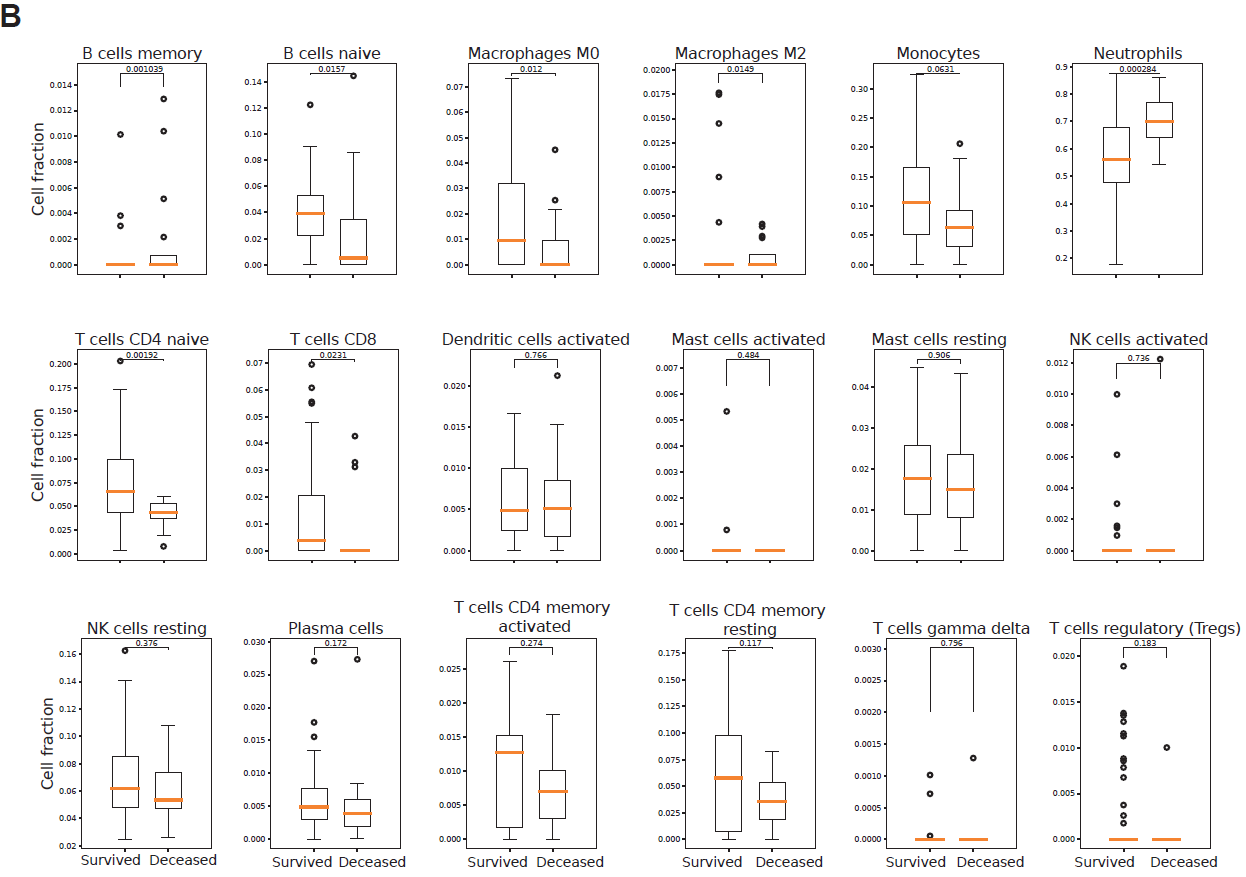


***Supplementary figure 7: Predicted immune cell type differences in patients with COVID-19, who either survived or died, and patients with influenza.*** *A) M0 macrophages, resting natural killer (NK) cells, plasma cells, cytotoxic CD8+ T cells and regulatory T cells were found to be significantly higher in COVID-19 patients. In influenza patients a significantly higher proportion of activated dendritic cells and M2 macrophages were detected. No significant difference was detected for naïve and memory B cells, M1 macrophages, activated and resting mast cells, monocytes, neutrophils, activated NK cells, activated or resting or naïve CD4+ memory T cells, and gamma delta T cells. B) A statistically significant higher count of neutrophils in COVID-19 patients who died after 30 days indicating the presence of an elevated innate immune response. Memory B cells were also significantly increased in COVID-19 patients who died. While an adaptive immune response was detected in COVID-19 survivors as can be seen by the statistically significant higher count of naïve B cells, and CD4+ and CD8+ T cells. Furthermore, M0 and M2 macrophages were significantly increased in patients who survived COVID-19. There was not significant difference detected for monocytes, activated dendritic cells, activated and resting mast cells, activated and resting NK cells, plasma cells, activated and resting memory CD4+ T cells, gamma delta T cells, and regulatory T cells.*


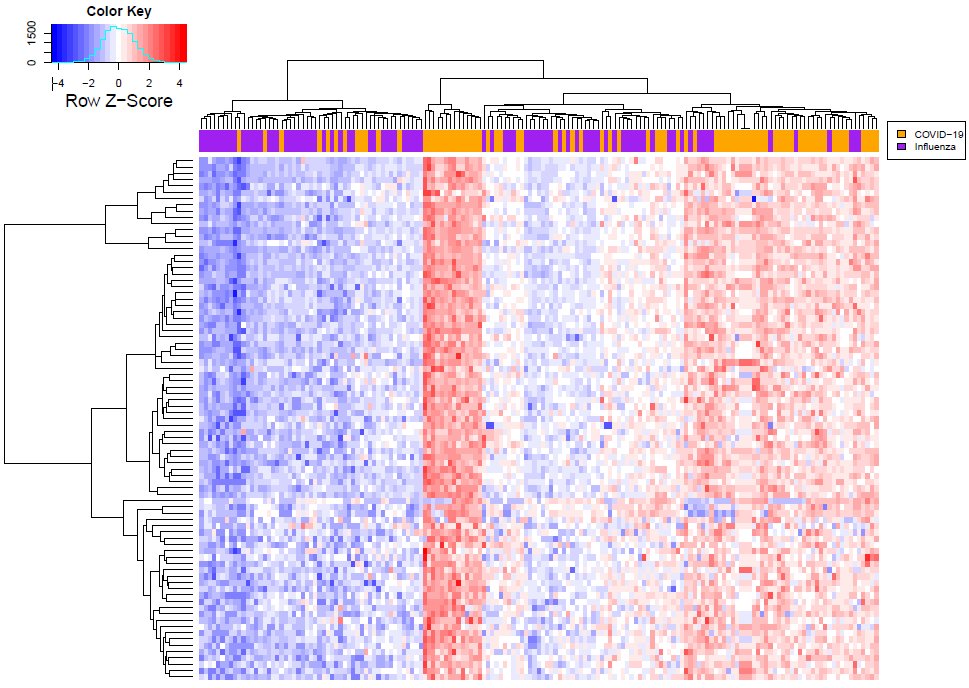


***Supplementary figure 8:*** ***Heatmap of 83 immunoglobulin gene transcripts associated with the GO biological process term ‘adaptive immune response’.*** *83 immunoglobulin genes, associated with an adaptive immune response, were found at a higher abundance in patients with COVID-19 based on positive Z-scores mostly in patients with COVID-19 while negative Z-scores are mostly seen in patients with influenza.*


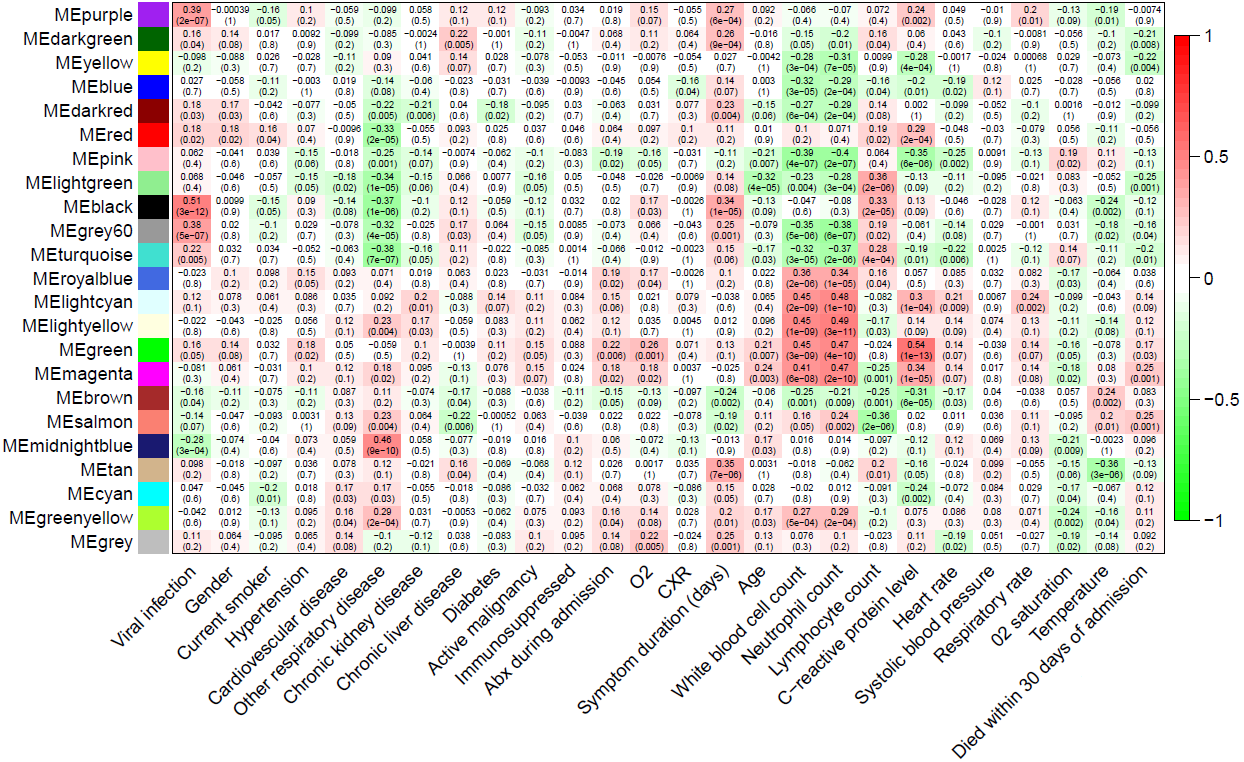


***Supplementary figure 9: : Clinical covariates and their correlation with different gene transcript clusters.*** *Weighted correlation network analysis was performed to assess the correlation between different clinical covariates and the expression of specific gene transcript clusters. These gene transcript clusters underwent GO analysis which revealed the associated biological process terms. The GO biological process terms are: blood coagulation (1.74x10^-22^, MEpurple), negative regulation of glycogen (starch) synthase activity (1.01x10^-02^, MEdarkgreen), erythrocyte development (1.41x10^-08^, MEyellow), neuron differentiation (1.16x10^-42^, MEblue), mRNA processing (2.85x10^-03^, MEdarkred), leukocyte granulation (1.26x10^-13^, MEred), immune effector process (2.83x10^-05^, MEpink), B cell activation (2.40x10^-09^, MElightgreen), complement activation classical pathway (1.48x10^-65^, MEblack), cellular response to interleukin-13 (1.88x10^-02^, MEgrey60), ncRNA metabolic process (1.22x10^-64^, MEturquiose), RNA processing (2.05x10^-32^, MEroyalblue), Fc-gamma receptor signaling pathway involved in phagocytosis (8.18x10^-05^, MElightcyan), no GO biological process terms detected (MElightyellow), neutrophil degranulation (1.27x10^-18^, MEgreen), myeloid leukocyte activation (3.66x10^-21^, MEmagenta), defense response to virus (6.88x10^-38^, MEbrown), purine ribonucleotide triphosphate metabolic process (1.41x10^-10^, MEsalmon), positive regulation of chemokine production (6.85x10^-04^, MEmidnightblue), mononuclear cell differentiation (2.57x10^-02^, MEtan), no GO biological process terms detected (MEcyan), reegulation of interleukin-6 production (4.10x10^-02^, MEgreenyellow), and no GO biological process terms detected (MEgrey).*


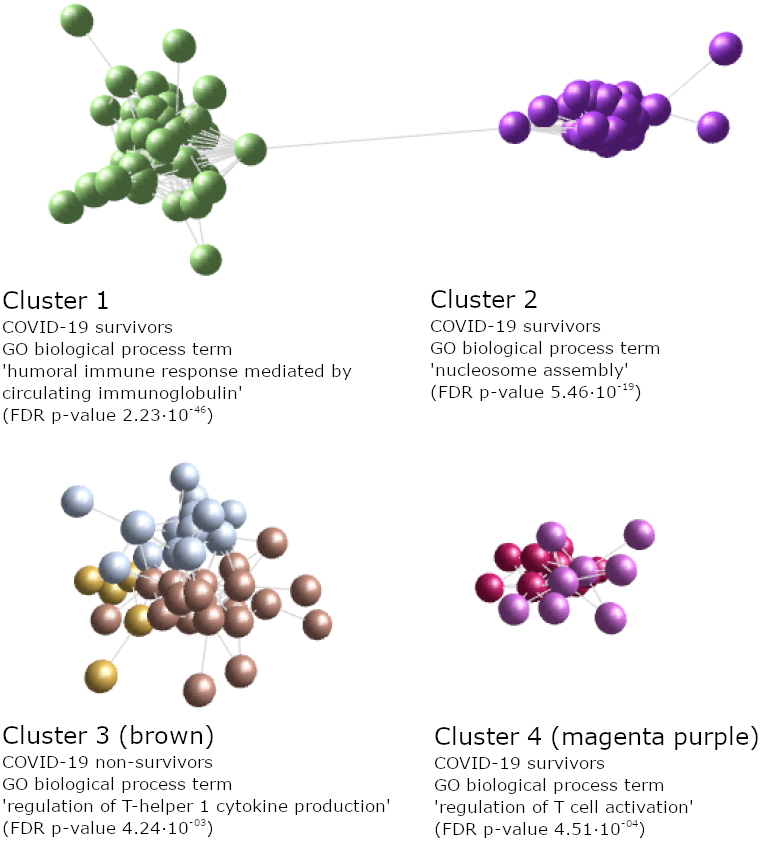


**Supplementary figure 10: Top 4 clusters identified between COVID-19 survivors and non-survivors.** For each cluster the predicted Gene Ontology biological process term identified by ToppGene are given, together with enrichment found in either COVID-19 survivors or non-survivors. From these top 4 clusters combinations of 100 genes were selected as potential predictor variables of COVID-19 outcome using Boosted Logistic Regression, Bayesian Generalised Linear and RandomForest models.
